# Cost-Effectiveness of Radar Localisation Versus Wire Localisation for Wide Local Excision of Non-palpable Breast Cancer

**DOI:** 10.1245/s10434-024-15142-x

**Published:** 2024-03-12

**Authors:** Chu Luan Nguyen, Rebecca Cui, Michael Zhou, Fatema Ali, Neshanth Easwaralingam, Belinda Chan, Susannah Graham, Farhad Azimi, Cindy Mak, Sanjay Warrier

**Affiliations:** 1 Department of Breast Surgery, Chris O’Brien Lifehouse, Camperdown, NSW Australia; 2 Department of Surgery, Royal Prince Alfred Hospital, Camperdown, NSW Australia; 3 Department of Surgery, The University of Sydney, Camperdown, NSW Australia

**Keywords:** Radar localisation, SAVI SCOUT, Wire localisation, Wide local excision, Breast cancer, Cost analysis

## Abstract

**Background:**

Wire localisation (WL) is the “gold standard” localisation technique for wide local excision (WLE) of non-palpable breast lesions but has disadvantages that have led to the development of wireless techniques. This study compared the cost-effectiveness of radar localisation (RL) to WL.

**Methods:**

This was a single-institution study of 110 prospective patients with early-stage breast cancer undergoing WLE using RL with the SCOUT^®^ Surgical Guidance System (2021–2023) compared with a cohort of 110 patients using WL. Margin status, re-excision rates, and surgery delays associated with preoperative localisation were compared. Costs from a third-party payer perspective in Australian dollars (AUD$) calculated by using microcosting, break-even point, and cost-utility analyses.

**Results:**

A total of 110 WLEs using RL cost a total of AUD$402,281, in addition to the device cost of AUD$77,150. The average additional cost of a surgery delay was AUD$2318. Use of RL reduced the surgery delay rate by 10% (*p* = 0.029), preventing 11 delays with cost savings of AUD$25,496. No differences were identified in positive margin rates (RL: 11.8% vs. WL: 17.3%, *p* = 0.25) or re-excision rates (RL: 14.5% vs. WL: 21.8%, *p* = 0.221). In total, 290 RL cases are needed to break even. The cost of WLE using RL was greater than WL by AUD$567. There was a greater clinical benefit of 1.15 quality-adjusted life-years (QALYs) and an incremental cost-utility ratio of AUD$493 per QALY favouring RL.

**Conclusions:**

Routine use of RL was a more cost-effective intervention than WL. Close to 300 RL cases are likely needed to be performed to recover costs of the medical device.

*Clinical trial registration*: ACTRN12624000068561.

**Supplementary Information:**

The online version contains supplementary material available at 10.1245/s10434-024-15142-x.

Breast cancer is one of the most common malignancies in women in Australia.^1^ Non-palpable breast cancers contribute a significant proportion of up to 35% of all new diagnoses of breast cancer.^2,3^ Successful surgical excision of non-palpable breast lesions relies on accurate preoperative localisation. Wire localisation (WL) has been the “gold standard” technique since its introduction in the 1970s, being widely used, effective, and relatively affordable.^4–6^ WL involves percutaneous insertion of a hooked wire into the lesion under imaging guidance.^2,5^ There are however limitations with this method, including patient discomfort, wire transection, and migration. Scheduling of WL usually is on the day of surgery to mitigate risk of migration, which can negatively impact theatre efficiency because of coupling of radiology and surgery scheduling.^6^

These limitations have led to the development of wireless localisation techniques, including SCOUT^®^ radar localisation (RL), which involves insertion of a tiny electromagnetic wave reflector into the target tissue by using an introducer needle delivery system under mammogram or ultrasound guidance. The reflector is activated by infrared light impulses generated by the console probe and uses two antennae to reflect an electromagnetic wave signal back to the handpiece to provide real-time directionality and proximity information.^7^

Evaluation of a new technology in regard to both its efficacy and associated costs can help with the decision to adopt it into clinical practice. RL has been shown to be a potential alternative to WL in small cohort studies, but data on the costs associated with it are lacking.^2,3,8–11^ This study compared the cost-effectiveness of RL to WL for wide local excision (WLE) of non-palpable breast tumours, in terms of margin status, re-excision rates, and surgery delays associated with preoperative localisation.

## Methods

### Study Design and Participants

This was a single-institution study of prospective patients with early breast cancer undergoing WLE using RL from February 2021 to April 2023. It was registered on the Australian New Zealand Clinical Trials Registry (ACTRN12624000068561) and followed the Consolidated Health Economic Evaluation Reporting Standards 2022 (CHEERS 2022) guidelines.^12^ Inclusion criteria were female patients ≥18 years of age with a non-palpable breast lesion(s) (≤2 cm on preoperative imaging) that underwent WLE. Lesions >2 cm in size were excluded because of treatment variability, including neoadjuvant chemotherapy, which could potentially alter tumour size for targeting and final specimen volume.^3^ Exclusion criteria were current pregnancy or lactation, previous breast or axillary surgery, previous neoadjuvant chemotherapy, or known allergy to components of SCOUT, such as nickel. Patients who met inclusion criteria underwent WLE by using SCOUT RL (Supplementary material). This cohort was compared to a retrospective cohort of consecutive patients, meeting the same inclusion criteria (except for allergy to components of SCOUT), who underwent WLE using standard WL.

### Surgical Technique

**Fig. 1:**
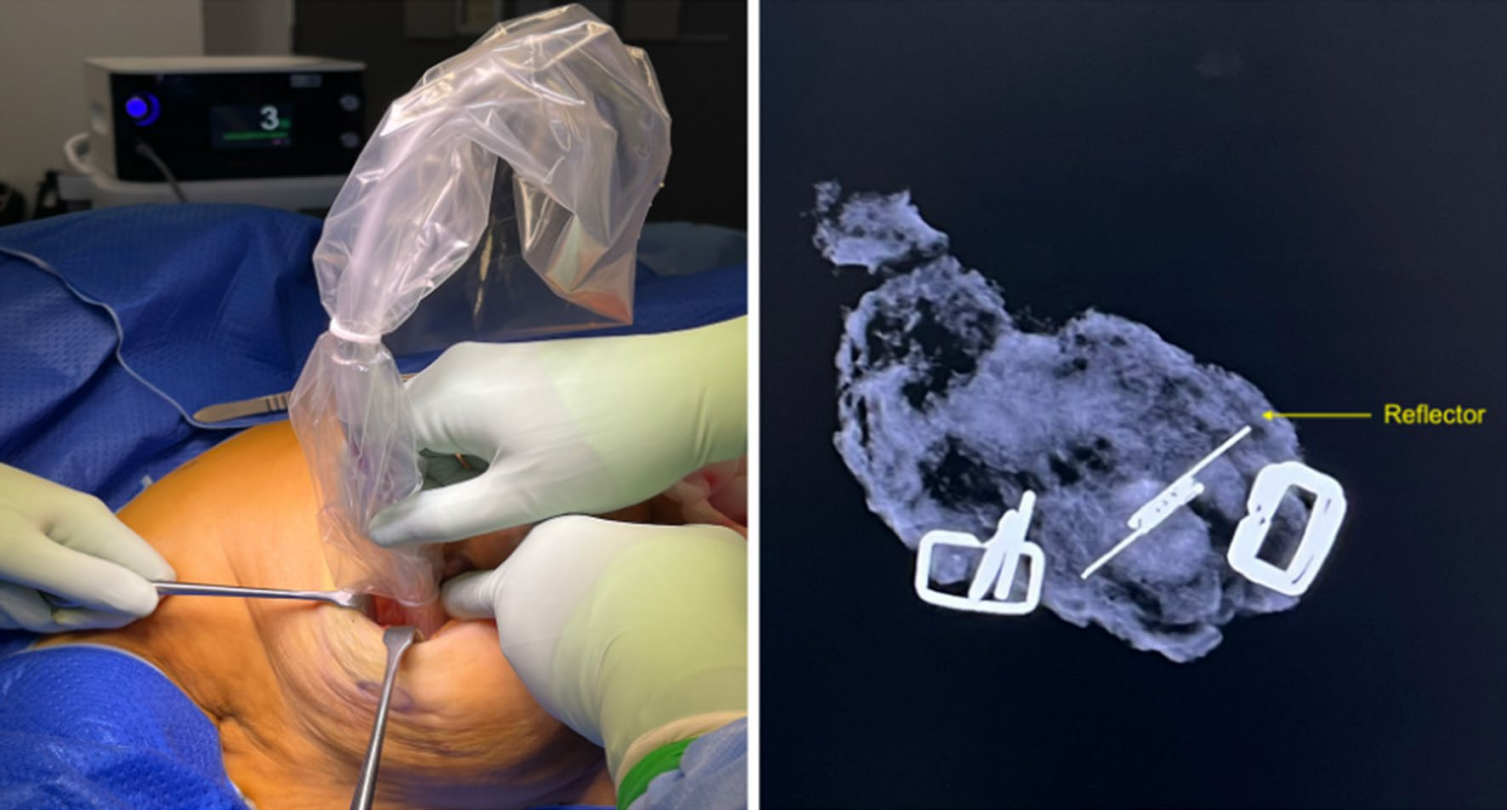
Left image: Radar localisation using the SCOUT Surgical Guidance System, with handpiece and integrated console, for wide local excision; Right image: Radiograph of the specimen with reflector in situ postoperative

Radar localisation involved the SCOUT Surgical Guidance System (Merit Medical Systems, Inc. South Jordan, UT), a radar reflector and an infrared light-emitting probe that guides the surgeon to the lesion. It consists of a 12-mm fiducial reflector preloaded into a 16-gauge introducer needle, and a console and handpiece system, which localises the reflector. The reflector contains an infrared light receptor, transistor switch, and two nitinol antennae. The handpiece generates an audible signal when positioned over the reflector. Preoperative localisation involved percutaneous insertion of the reflector into the breast after local anaesthesia and under ultrasound guidance by an interventional breast radiologist. The reflector was deployed adjacent to or within the targeted lesion or adjacent to the clip. Post-procedure mammogram confirmed satisfactory placement of the reflector (Fig. 1).^2,3,13–16^

Wire localisation involved the Kopans wire, a stainless steel, 0.3-mm diameter wire with a proximal reinforced portion and a spring hook to anchor to the tissue surrounding the lesion. Preoperative localisation involved a planning ultrasound to locate the lesion. After local anaesthesia, and under ultrasound guidance, the hookwire was advanced through the lesion and the needle deployed by an interventional breast radiologist. Four to 6 cm of the wire was left to protrude from the skin, and a mammogram was used to confirm the position following the procedure.^17^

Wide local excision was performed in the usual manner with a periareolar, inframammary, radial, or semicircular incision depending on the tumour location and wire entry point for WL cases. Dissection was performed with a combination of diathermy and scissors to excise the lump with an approximately 1-cm cuff of normal tissue to aim for clear margins. Dissection was guided by using audible cues from the SCOUT probe for the RL cases and by using the hookwire for the WL cases. The specimen was orientated, marked with sutures or clips, and examined macroscopically and with x-ray to confirm the lesion within the specimen. Local anaesthesia was given, and a layered closure was performed.

### Histopathology Assessment

Specialist breast pathologists provided histopathology reports, including surgical pathology and margin status. “Positive margins” and “close margins” were defined as tumour on ink and tumour ≤2 mm from ink, respectively, for invasive and in situ pathology.

### Outcomes

The study primary outcomes were margin status, re-excision rates, and surgery delays related to preoperative localisation. Theatre delay averted was the measure of effectiveness as it had significant implications on resource use. Other outcomes were tumour specimen weight and volume between RL and WL techniques. Demographic information included age, American Society of Anesthesiologists Classification (ASA) status, body mass index (BMI), and previous surgery or neoadjuvant therapy. The number of days before surgery that the reflector or hookwire was inserted, and any complication during and after insertion were recorded. Operative duration, length of hospital stay, and complications up to 90 days postoperative were documented. Characteristics of the tumour specimen included tumour type, grade, size, weight and volume, ER/PR/HER2/Ki67 status, histologic type, margin status, and re-excision rates.

### Cost Analysis

All costs were in 2023 Australian dollars (AUD$, Supplemental Table). The perspective of a “third-party” payer (Medicare), also known as a “healthcare payer” perspective, was adopted. A break-even point analysis was performed by using the equation: *“Number of cases to break-even” = “Fixed Cost”/ (“Savings per case” − “Variable cost”)*.^18,19^ The fixed cost was the purchase price of the SCOUT Surgical Guidance System (AUD$77,150). The variable cost was the cost of each use of SCOUT, including the reflector and delivery unit (AUD$498), and for each use of WL, including the hookwire (AUD$61.50). The savings per case was calculated by using: “*Savings per case” = “Average surgery delay cost” x “Surgery delay reduction rate.”* The cost of surgery delay because of preoperative localisation scheduling was calculated based on average costs of time (minutes) wasted in theatre by using microcosting, including costs for personnel and consumables related to each technique. The surgery delay reduction rate was the difference in rate of surgery delay between the RL and WL cohorts.^18,20^

Cost-utility analysis measures included quality-adjusted life-years (QALYs), which is the metric for both quality and quantity of life. Quality of life is determined by a patient’s preference towards a given health outcome (“health state”).^21^ QALYs provide a common unit of measurement that allows comparison of the value and clinical effectiveness of surgical interventions, which each have their own utility scores. Utility measures the success or failure of a surgical intervention based on how well the intervention impacts a patient’s final health outcome.^18,22^ A successful localisation for WLE and a surgery delay were defined as distinct “health states” with associated costs, probabilities, and utilities for use in the decision model. The costs for preoperative localisation and WLE were based on Medicare item numbers. Probabilities associated with health states were obtained for surgery with RL and with WL. Utilities used were obtained from surveys of surgeons.^23^ These are ratings of their preference for a given health state on a scale of 0 to 1, where 0 equals death and 1 equals perfect life. The utilities were converted to QALYs for all health states: *“QALY” = (“Utility of health state”) x (“Duration of health state”) + (“Utility of successful procedure”) x (“Remaining life years”), where “Remaining life years” = “Average life expectancy” − “Average age of patient,” and “Duration of health state” = “Estimated recovery time in weeks”/52 weeks.*^18,21^ The remaining life years value was calculated from the estimation that a female patient undergoing WLE is 59 years old and has a life expectancy of 85 years based on Australian Institute of Health and Welfare statistics.^1^ For a successful operation, defined as a successful localisation for WLE resulting in clear margins, the duration of health state was 0 while the duration for a delay in surgery was 1 day (1/365 years).

**Fig. 2:**
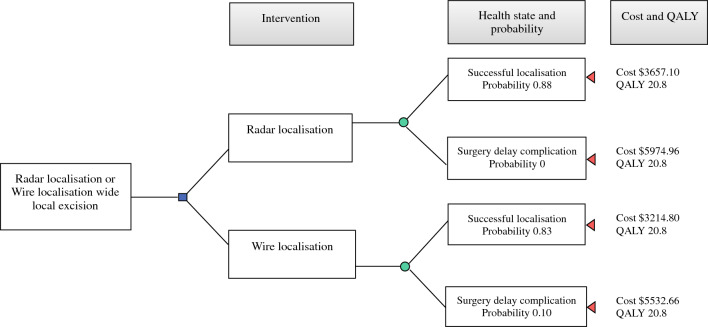
Markov model decision analytic tree diagram to analyse cost-effectiveness of wide local excision using radar localisation versus wire localisation. Probabilities are below each health state, and the total costs (AUD$) and quality-adjusted life-years (QALYs) are at the end. Blue square, decision node; Green circle, chance node; Red triangle, terminal node

A decision model was created for these data and incorporated the costs, QALYs and probabilities of the health states (Fig. 2). “Expected values” for costs and QALYs were calculated by multiplying the probability of a health state by its actual cost and QALYs, respectively. The incremental cost-utility ratio (ICUR) was calculated by using: *“ICUR” = (“Expected cost of RL” − “Expected cost of WL”) / (“Expected QALY of RL” − “Expected QALY of WL”)*.^21^ This ratio represents the added cost to extend a patient’s life by 1 year of perfect health. An intervention is “cost-effective” if the ICUR is greater than 0 and less than the “willingness to pay” for an added year of perfect health, which was defined as AUD$50,000.^21,22,24^ A one-way sensitivity analysis was performed to evaluate the robustness of the baseline decision analysis by observing the effect on the ICUR as the complication rate with use of WL was varied from 0 to 1.

### Statistical Analysis

Continuous variables were presented as means with standard deviation (SD) or as median with interquartile ranges (IQR) and dichotomous and categorical data as frequencies with percentages. The *t*-test was used to compare the mean for continuous variables between the two groups. A chi-squared test was used to compare the proportions of categorical variables between the two groups. Results with *p* ≤ 0.05 were considered statistically significant. Statistical analysis was performed with RStudio, v2023.09.2.

## Results

### Demographics

A total of 220 patients that underwent WLE for non-palpable breast lesions were included in this study. The mean age was 59.4 years (standard deviation [SD] 11.3) and most (72.7%) had invasive ductal carcinoma on final histopathology. A total of 110 patients had preoperative localisation using RL and 110 patients had it with WL. There were no significant differences in demographics and tumour characteristics of patients in the RL and WL cohorts (Table 1).

**Table 1:** Demographic and tumour characteristics of SCOUT^®^ radar and wire localisation cohorts for wide local excision of non-palpable breast lesions

	Radar localisation *n* = 110	Wire localisation *n* = 110	*p*
Age, mean (SD)	57 (11.5)	61.8 (10.7)	0.061
ASA grade, median (IQR)	2 (2, 2)	2 (2, 2)	0.545
BMI, mean (SD)	26 (3.6)	25.8 (3.6)	0.679
*Tumour characteristics, n* (%)			0.72
ER+	95 (86.4)	95 (86.4)	
PR+	87 (79.1)	89 (80.9)	
HER2+	18 (16.4)	15 (13.6)	
Ki67 ≥14%	48 (43.6)	48 (43.6)	
Triple negative	5 (4.5)	8 (7.3)	
*Pathology, n* (%)			0.906
IDC	77 (70)	83 (75.5)	
ILC	9 (8.2)	9 (8.2)	
DCIS	15 (13.6)	11 (10)	
Other	6 (5.5)	5 (4.5)	
Mixed^a^	3 (2.7)	2 (1.8)	
*Tumour grade, n (%)*			0.526
1	27 (24.5)	27 (24.5)	
2	53 (48.2)	47 (42.7)	
3	15 (13.6)	21 (19.1)	
Tumour size, mean mm (SD)	16.3 (14)	17.2 (13.2)	0.665
Tumour size, median mm (IQR)	12 (8, 17)	13 (7, 15)	

*ASA* American Society of Anesthesiologists, *BMI* body mass index, *DCIS* ductal carcinoma in situ, *ER+* estrogen receptor positive, *HER2+* human epidermal growth factor receptor 2 positive, *IDC* invasive ductal carcinoma, *ILC* invasive lobular carcinoma, *IQR* interquartile range, *PR+* progesterone receptor positive, *SD* standard deviation

^a^More than one different type of invasive cancer

### Preoperative Localisation

Placement of the reflector for RL was performed a median 3 days before the day of surgery (range 1–20 days), whereas patients who had WL underwent hookwire placement the day of surgery (range 0–1 days). No significant issues were identified during insertion and localisation of the RL cases. There were four cases encountered during the hookwire insertion, which all involved the hookwire dislodging immediately after insertion. This was identified on the post-procedure mammogram, and a second hookwire placed successfully in all cases.

### Surgical Outcomes

Median specimen weights were similar between the RL and WL cohorts (RL 27 g [IQR 15.4–39.8] versus WL 30.7 g [IQR 19.3–53], *p* = 0.454, respectively). Median specimen volumes were similar between the RL and WL cohorts (RL 50.4 cc [IQR 30–79.5] versus WL 60 cc [IQR 34–108], *p* = 0.377, respectively). Positive margin, close margin, and reexcision rates for the RL cohort were 11.8%, 32.7%, and 14.5%, respectively. The WL cohort was not significantly different with regard to positive margin, close margin, and re-excision rates of 17.3%, 34.5%, and 21.8%, respectively (Table 2). In the RL cohort, there were two superficial surgical site infections, which were managed conservatively with antibiotics: two haematomas of which one required evacuation in theatre, and one pneumonia treated with antibiotics. In the WL cohort, there were two haematomas, one of which required evacuation in theatre and two seromas both managed conservatively.

**Table 2:** Surgical outcomes of SCOUT^®^ radar and wire localisation cohorts for wide local excision of non-palpable breast lesions

	Radar localisation *n* = 110	Wire localisation *n* = 110	*p*
Insertion before surgery, median days (range)	3 (1, 20)	0 (0, 1)	**<0.001**
Tumour weight, median g (IQR)	27 (15.4, 39.8)	30.7 (19.3, 53)	0.454
Tumour volume, median cc (IQR)	50.4 (30, 79.5)	60 (34, 108)	0.377
Positive margin, *n* (%)	13 (11.8)	19 (17.3)	0.25
Close margin^a^, *n* (%)	36 (32.7)	38 (34.5)	0.569
Re-excision, *n* (%)	16 (14.5)	24 (21.8)	0.221
Surgery delay^b^, *n* (%)	0	11 (10%)	**0.029**
Theatre duration, mean mins (SD)	74.7 (27.6)	60 (17.3)	0.052
Hospital stay, median days (IQR)	0 (1, 1)	0 (1, 1)	0.182
*Complications, n* (%)			1
Localisation related	0	4 (3.7)	
Other^c^	5 (4.5)	4 (3.6)	

SD standard deviation; IQR interquartile range

^a^Close margin defined as tumour ≤2 mm from ink

^b^Related to preoperative localisation

^c^Surgical site infection, haematoma, seroma

Bold *p* values represent statistically significant results

### Cost Analysis

The surgery delay rate in the RL cohort was significantly less compared with the WL cohort (0% vs. 10%, respectively; *p* = 0.029; Table 2). These delays were related to preoperative localisation and were logistical issues because of scheduling of radiology and surgery. The overall cost of RL utilisation over the 2-year study period was AUD$402,281 AUD, which included the cost of each RL use and the reflector. The cost of a single use of RL per case was AUD$3657.10. The average cost of a surgery delay in the WL cohort was AUD$2317.86 due to a mean delay of 79.1 min (SD 50.2). Routine use of RL reduced the surgery delay complication rate by 10% and prevented such delays in 11 cases resulting in a gross cost savings of AUD$25,496.46 (Table 3).

**Table 3:** Costs of SCOUT^®^ radar localisation for wide local excision

	Cost (AUD$)
*Surgery delay complication, n = 0*	
Average surgery delay cost^a^	2317.86
Average cost of WLE using RL	3657.10
Total costs	5974.96
*Other institution costs*^b^	
Single use of reflector	498.00
Probe disposable cover	5.80
Theatre room fee (per min)	15.80
Surgeon/ anaesthetist fee (per min)	3.54
Other personnel fee (per min)^c^	10.00
Gross savings (11 delays saved)	25,496.46
Total radar localisation cost (*n* = 110)	402,281

^a^Mean 79-min surgery delay

^b^Full list of costs for other consumables used for operations listed in the supplemental material

^c^Includes theatre scrub and anaesthetic nursing staff

*AUD$* Australian dollars, *WLE* wide local excision, *RL* radar localisation

The break-even point analysis demonstrated that 290 cases using RL are needed to be performed routinely to recover costs of the medical device based on the average surgery delay cost of AUD$2317.86 and a surgery delay reduction rate of 10%. The greater the number of cases performed with RL, the greater the potential savings when this break-even point is overcome. Baseline cost-utility analysis showed that although the cost of WLE with RL was greater than that with WL by AUD$566.92, use of RL yielded a greater clinical benefit of 1.15 QALYs and the ICUR was only AUD$492.97 (Table 4). The one-way sensitivity analysis demonstrated that using RL was more cost-effective when the surgery delay complication rate with WL was ≥10% (using a maximum willingness to pay of AUD$50,000 per QALY).

**Table 4:** Utilities, costs, and quality-adjusted life-years for wide local excision using SCOUT^®^ radar localisation versus wire localisation

Health state	Utility	Probability	Cost, AUD$	Expected Cost, AUD$^a^	QALY	Expected QALY^a^
*Radar localisation*						
Successful localisation^b^	0.8	88.2	$3657.10	$3225.56	20.8	18.35
Surgery delay complication	0.75	0	$5974.96	$0	20.8	0
*Wire localisation*						
Successful localisation^b^	0.8	82.7	$3214.80	$2658.64	20.8	17.2
Surgery delay complication	0.75	0.10	$5532.66	$553.266	20.8	2.08
*Baseline cost-analysis*						
Baseline cost difference		$566.92				
QALY difference		1.15				
Incremental cost-utility ratio (ICUR)		$492.97				

*AUD$* Australian dollars, *QALY* quality-adjusted life-years

^a^Expected cost and QALY calculated based on probability and actual costs

^b^Successful localisation defined as no margin positivity

## Discussion

This is the first published study to date that assessed both surgical outcomes and costs between RL and WL in WLE for early-stage breast cancer.^25^ The results support previous findings that there was no significant difference in surgical outcomes between RL and WL techniques.^2,3,8–11^ It also demonstrated that WLE using RL was a cost-effective technique for reducing delays in surgery related to preoperative localisation with WL.

The standard technique for locating non-palpable breast lesions with WL has well-documented disadvantages. The wire entry point often is chosen based on ease of access as opposed to an ideal surgical approach. The surgeon must also estimate the pathway to the lesion, potentially leading to inaccuracy in locating the targeted lesion. Wire migration can cause excision of excessive tissue, and wire transection can leave fragments within the breast.^6^ Findings also have shown that WL can be relatively inaccurate in achieving clear margins, with re-excision rates reported as high as 60%.^26,27^ Specimen resection weights and volumes, as well as rate of positive margin, close margin and re-excision rates between the RL and WL cohorts in this current study were equivalent. The positive margin rate of 11.8% and re-excision rate of 14.5% were comparable to those reported in other studies (Table 5).^2,3,8–11,13^

**Table 5:** Studies comparing SCOUT^®^ radar localisation with wire localisation for wide local excision of non-palpable breast lesions

Study, year	Patients (RL, WL), *n*	Study type	RL positive margin, *n* (%)	RL re-excision, *n* (%)	WL positive margin, *n* (%)	WL re-excision, *n* (%)
Patel, 2018 ^3^	84, 42	S, R	4 (9.5)	3 (7.1)	3 (7.1)	4 (9.5)
Tingen, 2020^10^	320, 175	S, R	18 (5.6)	17 (5.3)	24 (13.7)	24 (13.7)
Bercovici, 2021^8^	202, 123	S, R	17 (8.4)	Not reported	17 (13.8)	Not reported
Choe, 2022 ^9^	254, 352	S, R	48 (18.9)	Not reported	60 (17)	Not reported
Farha, 2023 ^11^	64, 48	S, R	Not reported	1 (1.6)	Not reported	4 (8.3)
Current study, 2023	110, 110	S, P	13 (11.8)	16 (14.5)	19 (17.3)	24 (21.8)

*RL* radar localisation, *WL* wire localisation, *S* single institution, *R* retrospective, *P* prospective, *SD* standard deviation, *IQR* interquartile range

Radar localisation was developed with the goal to overcome the disadvantages of standard techniques for locating non-palpable breast lesions. The reflector can be placed from any direction without impacting the surgical approach, allowing for optimal incision placement. The localisation technique provides real-time dynamic feedback during the dissection, allowing for continual correction by the surgeon to afford accurate removal of the reflector and lesion while minimising excessive tissue removal. In this study, the handpiece guided the entire dissection from the skin surface in some cases. In cases where the reflector was not detected from the skin surface, the approximate reflector location was able to be estimated from the postinsertion images. This allowed for successful localisation and removal of all reflectors. The previously reported issues with the radar being disabled by electrocautery equipment and radar displacement were not noted in this current cohort.^8^

Hookwires often need to be placed on the day of the operation, directly coupling surgery and radiology schedules, which can inconvenience the patient, radiologist, surgeon, and theatre staff. The ability to place the reflector several days or even weeks before surgery helps eliminate scheduling conflicts between radiology and theatre. This uncoupling of radiology and theatre schedules, in contrast to WL, has the potential to improve theatre efficiency by mitigating the risk of delays related to preoperative localisation.^28^ This increased flexibility and ease of surgical scheduling was observed in the RL cohort in this current study who had the radar placed a median of 3 days before surgery compared with day before or day of surgery in the WL cohort.^2,8^

Radar localisation surveys completed by participating surgeons have been previously reported. RL was rated better than WL for incision site planning, tissue localisation, confidence in removing the correct target, as well as the ability to start cases earlier, and reduction in theatre schedule delays. Surveys of patients’ experiences with RL also showed good satisfaction with the preoperative localisation technique.^7^ This potential advantage of increased patient comfort was observed within the RL cohort of this current study. There were no patients that required reinsertion of the reflector compared with four patients who required a second procedure for misplacement or migration of the hookwire, although not statistically significant.^2,3^ Further studies assessing patient reported outcomes are needed to further validate this.

The costs involved with a new technology compared to current “gold standard” is a crucial consideration in its adoption.^25^ Compared with most wireless techniques, WL is economical.^29–31^ RL requires additional expenditure, including consideration of upfront costs of the console and guidance probe. This study found that RL with SCOUT was more expensive than standard WL. The fixed cost for RL use included the imaging guidance system (total, AUD$77,150), whereas WL did not require any initial outlay cost. The ongoing costs associated with RL were also more expensive than WL. The economic advantage of RL over WL found was the result of the 10% reduction in number of delays in theatre because of logistical issues with preoperative localisation. Although the cost of WLE with RL was greater than WL by AUD$567, use of RL ended up yielding a greater clinical benefit of 1.15 QALYs by reducing the rate of surgery delays. In addition to this, the break-even point analysis demonstrated that 290 cases using RL need to be performed routinely to recover costs of the initial outlay costs for the medical device. This suggested that RL was a cost-effective technique, and the cost savings could potentially be even greater in the long-term when the break-even point is overcome.

Investment in this newer method could be justified as the additional cost involved in using RL was associated with reducing costs related to delays in surgery from preoperative localisation. There was no significant difference in surgical outcomes between the two techniques, making them equivalent in terms of efficacy, but delays in surgery were found to be costly. There were multiple other variables not evaluated in this analysis because of difficulty quantifying their costs, such as avoidance of distress and trauma of a hookwire for the patient, potential costs of clips at the time of biopsy followed by a second radiology visit for the hookwire insertion, and costs to radiology because of any delays in their schedule. The main drawback with WL is related to the requirement for performing WL on the day before or same day of surgery. This scheduling coupling between localisation procedure and surgery has already been shown to negatively impact workflow; it excludes such cases from being the first operative case of the day and can delay surgery start times, resulting in inefficiencies and their associated costs.^28,32^ Wireless localisation techniques have been shown to reduce operative and radiology delays.^33^ The trade-off for such wireless technologies, such as SCOUT, is the initial expense of equipment costs for the console and probe.^34,35^ Increased uptake of RL technology could potentially lower costs of the imaging system, which is a significant limiting factor in its adoption.

Limitations of this study include that the results may only be generalisable to healthcare systems with similar funding arrangements to the Australian system. The control cohort also was retrospective in nature. Data on multiple visits and distance travelled related to localisation procedure, and surgery were not available. The quality-of-life benefits associated with less hospital visits and travel were unable to be factored into this cost analysis. The “perspective” of the study determines whose costs will be included in the analysis and is important in drawing conclusions. This study was limited by a “healthcare payer” perspective, which included costs to a third party (Medicare). A “healthcare sector” perspective includes costs to the healthcare payer but also patients’ out-of-pocket costs. A “societal perspective” includes all these costs as well as loss of productivity to patients and carers.^22^ There are no trials to date comparing RL with WL, and large multi-institutional prospective trials that also evaluate economical aspects, including a more wholistic “societal” perspective, could help to validate the cost-effectiveness of RL

To optimise spending of scarce resources in healthcare, public and private payers need evidence of the added value of medical interventions before deciding on reimbursement. In this study, RL technology was found to be intuitive, reliable, and easy to implement. The surgeons favoured RL because of its ease of intraoperative localisation and simplified scheduling. Radar localisation is a reasonable alternative to WL, but this comes at an upfront dollar cost that must be considered. The upfront costs of RL, however, could be offset by cost savings due to decoupling of radiology and surgery schedules. Routine use of RL was a cost-effective intervention as the additional cost of RL was less than the cost of surgery delays with WL. Nearly 300 cases using RL are likely needed to be performed to recover costs of the device. This information could assist in the decision to adopt this technology in clinical practice.

### Supplementary Information

Below is the link to the electronic supplementary material.

Supplementary file1 (DOCX 15 kb)

Supplementary file2 (DOCX 69 kb)

## References

[1] Australian institute of health and welfare (aihw). Deaths in australia. https://www.aihw.gov.au/reports/life-expectancy-deaths/deaths-in-australia/contents/about. Accessed 20 Oct 2023.

[2] Kasem I, Mokbel K (2020). Savi scout(r) radar localisation of non-palpable breast lesions: Systematic review and pooled analysis of 842 cases. Anticancer Res..

[3] Patel SN, Mango VL, Jadeja P (2018). Reflector-guided breast tumor localization versus wire localization for lumpectomies: A comparison of surgical outcomes. Clin Imaging..

[4] Dodd G, Fry K, Delany W (1965). Pre-operative localization of occult carcinoma of the breast.

[5] Frank HA, Hall FM, Steer ML (1976). Preoperative localization of nonpalpable breast lesions demonstrated by mammography. N Engl J Med..

[6] Norman C, Lafaurie G, Uhercik M, Kasem A, Sinha P (2021). Novel wire-free techniques for localization of impalpable breast lesions-a review of current options. Breast J..

[7] Cox CE, Garcia-Henriquez N, Glancy MJ (2016). Pilot study of a new nonradioactive surgical guidance technology for locating nonpalpable breast lesions. Ann Surg Oncol..

[8] Bercovici N, Makarenko V, Vijayaraghavan G (2021). A single-institution analysis of reflector-guided localization using savi scout(r) in nonpalpable breast carcinoma compared to traditional wire localization. Breast J..

[9] Choe AI, Ismail R, Mack J (2022). Review of variables associated with positive surgical margins using scout reflector localizations for breast conservation therapy. Clin Breast Cancer..

[10] Tingen JS, McKinley BP, Rinkliff JM, Cornett WR, Lucas C (2020). Savi scout radar localization versus wire localization for breast biopsy regarding positive margin, complication, and reoperation rates. Am Surg..

[11] Farha MJ, Simons J, Kfouri J, Townsend-Day M (2023). Savi scout(r) system for excision of non-palpable breast lesions. Am Surg..

[12] Equator network. Reporting guidelines for main study types. https://www.equator-network.org/reporting-guidelines/. Accessed 25 Oct 2023.

[13] Mango VL, Wynn RT, Feldman S (2017). Beyond wires and seeds: Reflector-guided breast lesion localization and excision. Radiology..

[14] Dauphine C, Reicher JJ, Reicher MA (2015). A prospective clinical study to evaluate the safety and performance of wireless localization of nonpalpable breast lesions using radiofrequency identification technology. AJR Am J Roentgenol..

[15] Falcon S, Weinfurtner RJ, Mooney B, Niell BL (2018). Savi scout(r) localization of breast lesions as a practical alternative to wires: Outcomes and suggestions for trouble-shooting. Clin Imaging..

[16] Jadeja PH, Mango V, Patel S (2018). Utilization of multiple savi scout surgical guidance system reflectors in the same breast: A single-institution feasibility study. Breast J..

[17] Hall FM, Kopans DB, Sadowsky NL, Homer MJ (2013). Development of wire localization for occult breast lesions: Boston remembrances. Radiology..

[18] Nguyen CL, Dayaratna N, Comerford AP (2022). Cost-effectiveness of indocyanine green angiography in postmastectomy breast reconstruction. J Plast Reconstr Aesthet Surg..

[19] Nguyen CL, Zhou M, Easwaralingam N (2023). Novel dual tracer indocyanine green and radioisotope versus gold standard sentinel lymph node biopsy in breast cancer: The greenorblue trial. Ann Surg Oncol..

[20] Tedesco G, Faggiano FC, Leo E, Derrico P, Ritrovato M (2016). A comparative cost analysis of robotic-assisted surgery versus laparoscopic surgery and open surgery: The necessity of investing knowledgeably. Surg Endosc..

[21] Shauly O, Gould DJ, Patel KM (2020). Cost-utility analysis comparing surgical and nonsurgical interventions in the treatment of erectile dysfunction. Eur J Plast Surg..

[22] Vissapragada R, Bulamu N, Karnon J, Yazbek R, Watson DI (2021). Cost-effectiveness in surgery: Concepts of cost-utility analysis explained. ANZ J Surg..

[23] Yoon AY, Bozzuto L, Seto AJ, Fisher CS, Chatterjee A (2019). A systematic review of utility score assessments in the breast surgery cost-analysis literature. Ann Surg Oncol..

[24] Bertram MY, Lauer JA, De Joncheere K (2016). Cost-effectiveness thresholds: Pros and cons. Bull World Health Organ..

[25] Wright CM, Moorin RE, Saunders C (2021). Cost-effectiveness of radioguided occult lesion localization using 125i seeds versus hookwire localization before breast-conserving surgery for non-palpable breast cancer. Br J Surg..

[26] Gray RJ, Salud C, Nguyen K (2001). Randomized prospective evaluation of a novel technique for biopsy or lumpectomy of nonpalpable breast lesions: Radioactive seed versus wire localization. Ann Surg Oncol.

[27] Hughes JH, Mason MC, Gray RJ (2008). A multi-site validation trial of radioactive seed localization as an alternative to wire localization. Breast J..

[28] Murphy JO, Moo TA, King TA (2013). Radioactive seed localization compared to wire localization in breast-conserving surgery: Initial 6-month experience. Ann Surg Oncol..

[29] Kapoor MM, Patel MM, Scoggins ME (2019). The wire and beyond: Recent advances in breast imaging preoperative needle localization. Radiographics..

[30] Jeffries DO, Dossett LA, Jorns JM (2017). Localization for breast surgery: The next generation. Arch Pathol Lab Med..

[31] Corsi F, Sorrentino L, Bossi D, Sartani A, Foschi D (2013). Preoperative localization and surgical margins in conservative breast surgery. Int J Surg Oncol..

[32] Loving VA, Edwards DB, Roche KT (2014). Monte carlo simulation to analyze the cost-benefit of radioactive seed localization versus wire localization for breast-conserving surgery in fee-for-service health care systems compared with accountable care organizations. AJR Am J Roentgenol..

[33] Zhang Y, Seely J, Cordeiro E (2017). Radioactive seed localization versus wire-guided localization for nonpalpable breast cancer: A cost and operating room efficiency analysis. Ann Surg Oncol..

[34] Mayo RC, Kalambo MJ, Parikh JR (2019). Preoperative localization of breast lesions: Current techniques. Clin Imaging..

[35] Kalambo M, Parikh JR (2017). Implementing the savi scout system in community radiology practice. J Am Coll Radiol..

