# Supplementary material for: Cost-Effectiveness of Radar Localisation Versus Wire Localisation for Wide Local Excision of Non-palpable Breast Cancer
Source: Ann Surg Oncol. 2024 Mar 12;31(6):3916–25. doi: 10.1245/s10434-024-15142-x (PMC11076345; doi:10.1245/s10434-024-15142-x)
Supplement: Supplementary file 1 — Supplementary file1 (DOCX 15 kb) [file 10434_2024_15142_MOESM1_ESM.docx]

Supplemental Table. Costs of using SCOUT® radar localisation versus wire localisation for wide local excision of non-palpable breast lesions.

| **Item** | **Cost, AUD$** | |
| --- | --- | --- |
|  | **Radar localisation** | **Wire localisation** |
| **Initial outlay cost** |  |  |
| RL console^a^ | 70,000 |  |
| RL probe^a^ | 7150 |  |
| **Reflector/ hookwire** |  |  |
| Reflector & delivery needle per unit^a^ | 498.00 |  |
| Hookwire per unit^b^ |  | 61.50 |
| Local anaesthetic | 70.00 | 70.00 |
| **Theatre consumables** |  |  |
| Probe disposable sterile cover | 5.80 |  |
| **Theatre costs**^c^ |  |  |
| Surgeon/ anaesthetist fee (per min) | 3.54 | 3.54 |
| Other personnel fee^d^ (per min) | 10.00 | 10.00 |
| Theatre room fee (per min) | 15.80 | 15.80 |
| **Radiology costs (Medicare)** |  |  |
| Preoperative localisation of lesion | 427.25 | 427.25 |
| Radiologist physician fee (per min) | 2.04 | 2.04 |
| Other personnel fee^e^ (per min) | 1.02 | 1.02 |
| **Operation (Medicare)** |  |  |
| Wide local excision | 712.05 | 712.05 |
| **Total cost per case**^g^ | **3657.10** | **3214.80** |
|  |  |  |

AUD$, Australian dollars

^a^SAVI SCOUT Surgical Guidance System (Merit Medical, Victoria, Australia)

^b^Kopans

^c^Cost per median operation duration of 60 minutes

^d^Includes theatre scrub and anaesthetic nursing staff

^e^Cost per median localisation procedure duration of 60 minutes

^f^Includes radiolographer and nursing staff

^g^Not inclusive of initial outlay cost of imaging guidance system
