# Supplementary material for: Cost-Effectiveness of Radar Localisation Versus Wire Localisation for Wide Local Excision of Non-palpable Breast Cancer
Source: Ann Surg Oncol. 2024 Mar 12;31(6):3916–25. doi: 10.1245/s10434-024-15142-x (PMC11076345; doi:10.1245/s10434-024-15142-x)
Supplement: Supplementary file 2 — Supplementary file2 (DOCX 69 kb) [file 10434_2024_15142_MOESM2_ESM.docx]

Protocol Title: An Audit of Savi Scout ® Radar localisation for non-palpable breast lesions.

| Protocol Number | X23-0162 |
| --- | --- |
| Study Title | An Audit of Savi Scout ® Radar localisation for non-palpable breast lesions |
| Coordinating Principal Investigator | Dr Farhad Azimi |
| Signature: |  |
| Date: | 13/07/2023 |
| Protocol Authors (Co-investigators) | A/Prof Cindy Mak, A/Prof Sanjay Warrier, Dr Belinda Chan, Dr Susannah Graham |
| Other Individuals Involved in Study / Role | Mr Jack Kelly / Investigator  Ms RuYu (Ruth) Li / Medical Student  Ruth would be conducting the study to contribute to the requirements of Doctor of Medicine (University of Sydney) under the supervision of Dr Farhad Azimi. She would be involved in creating the Participant Information Sheet and Consent Forms for participant recruitment. |

**Ethics Statement:**

The study will be conducted in accordance with the *National Statement on Ethical Conduct in Human Research* (2007) ([Link to National Statement](https://www.nhmrc.gov.au/about-us/publications/national-statement-ethical-conduct-human-research-2007-updated-2018)) , the *CPMP/ICH Note for Guidance on Good Clinical Practice* ([Link to CPMP/ICH](https://www.tga.gov.au/publication/note-guidance-good-clinical-practice-july-2000) ) and consistent with the principles that have their origin in the Declaration of Helsinki. Compliance with these standards provides assurance that the rights, safety and well-being of trial participants are respected.

| **Protocol Title** | An Audit of Savi Scout ® Radar localization for non-palpable breast lesions |
| --- | --- |
| **Objectives** | The primary objectives of iBRA-net Study are:   - To evaluate the efficacy of localization of non-palpable breast lesions with Savi Scout ®. - To establish the efficacy of axillary node marking with Savi Scout ®.   The secondary objectives are:   - To compare the efficacy of Savi Scout ® with hookwire locations of non-palpable breast lesions. |
| **Study design** | Prospective Audit |
| **Planned sample size** | 1000 (worldwide), 50 (COBL) |
| **Selection criteria** | All patients over the age of 18 electing to undergo a breast conserving localization procedure or axillary nodal marking using the Savi Scout ® System will be eligible for inclusion. |
| **Study Procedure** | Potential participants will be identified prospectively by consultant surgeons. Simple demographic, procedure and process data will be contemporaneously collected for each participant. Data will be recorded in an anonymized format using a unique alphanumeric study identification number on a secure web-based database (REDCap) designed by Vanderbilt University and hosted by Manchester University NHS Foundation Trust/Manchester University. |
| **Statistical considerations** | Power Calculation - with n=1000 patients per group, the upper limit of the observed one-sided 95% confidence interval for the difference between failure rates (seed vs wire) is expected to be less than 0.9% with 80% power, assuming the two methods both have an expected failure rate of 0.6%. Hence, if a 0.9% difference is considered an acceptable equivalence margin (eg. 0.6% for wire and 1.5% for seed), 1000 patients per group should be sufficient to establish equivalence. Simple summary statistics will be calculated for each outcome and regression analysis used to control for predictive variables. Data will be tested for distribution and differences between groups using unpaired t-tests, Mann-Whitney U tests and Chi squared tests as appropriate, based on recruitment. |
| **Time Period of Data Collection** | May 2023 – May 2024 |
| **Duration of the Study** | 1 year |

**SUMMARY**

[if applicable:] Protocol Version Control box

| **Protocol**  **Version Number** | **Date** | **Summary of Changes** |
| --- | --- | --- |
|  |  |  |
|  |  |  |
|  |  |  |
|  |  |  |

**CONTENTS**

[**1.**  **BACKGROUND AND INTRODUCTION** 6](#_Toc117764224)

[1.1. DISEASE BACKGROUND 6](#_Toc117764225)

[1.2. RATIONALE FOR PERFORMING THE STUDY 6](#_Toc117764226)

[**2.** **HYPOTHESIS** 6](#_Toc117764227)

[**3.**  **STUDY OBJECTIVES / AIMS** 6](#_Toc117764228)

[3.1. PRIMARY OBJECTIVES 6](#_Toc117764229)

[3.2. SECONDARY OBJECTIVES 6](#_Toc117764230)

[**4.**  **STUDY DESIGN** 7](#_Toc117764231)

[4.1. DESIGN / STUDY TYPE 7](#_Toc117764232)

[4.2. EXPECTED PARTICIPANT NUMBERS 7](#_Toc117764233)

[4.3. TIME PERIOD OF THE STUDY 7](#_Toc117764234)

[4.4 ENDPOINTS 8](#_Toc117764235)

[4.5. CENTRES (STUDY SITES) 8](#_Toc117764236)

[**5.**  **STUDY PARTICIPANTS** 8](#_Toc117764237)

[5.1. INCLUSION CRITERIA 8](#_Toc117764238)

[5.2. EXCLUSION CRITERIA 9](#_Toc117764239)

[5.3 KEY ELEMENTS OF RECRUITMENT (AS PER NS) 9](#_Toc117764240)

[5.4 CONFOUNDERS 10](#_Toc117764241)

[5.5 STUDY LIMITATIONS 10](#_Toc117764242)

[**6.**  **STUDY PROCEDURES** 10](#_Toc117764243)

[6.1. INVESTIGATION PLAN 10](#_Toc117764244)

[6.2. INFORMATION AND CONSENT (OR WAIVER OF CONSENT) 10](#_Toc117764245)

[**7.** **OUTCOMES** 11](#_Toc117764246)

[7.1. DEFINITION OF OUTCOMES 11](#_Toc117764247)

[**8.**  **STATISTICAL CONSIDERATIONS** 11](#_Toc117764248)

[8.1. SAMPLE SIZE OR POWER CALCULATION 11](#_Toc117764249)

[8.2. PROVIDE A DETAILED ANALYSIS PLAN 11](#_Toc117764250)

[**9.** **DATA COLLECTION AND CONFIDENTIALITY AND STORAGE AND ARCHIVING OF STUDY** 11](#_Toc117764251)

[9.1. FORMS AND PROCEDURE FOR COLLECTING DATA – 11](#_Toc117764252)

[9.2. SYSTEMS 11](#_Toc117764253)

[9.3 RESEARCH DATA MANAGEMENT PLAN (RDMP) 12](#_Toc117764254)

[**10.** **ETHICS AND PROTOCOL AMENDMENTS** 12](#_Toc117764255)

[**11. PUBLICATION & INTELLECTUAL PROPERTY** 12](#_Toc117764256)

[**12. REFERENCES** 12](#_Toc117764257)

[**13.**  **APPENDICES** 12](#_Toc117764258)

### **1. BACKGROUND AND INTRODUCTION**

1.1. DISEASE BACKGROUND

Excision of impalpable breast lesions is usually directed by preoperative wire placement into or adjacent to the target lesion, although there is a move towards using more practical devices which require intra-operative detection, such as Iodine (^125^I) radioactive seed localization, Magseed® (detectable magnetic field), the SAVI Scout® (radar technology) and the LOCalizer™ Radiofrequency Identification (RFID) System. Wire localization presents several challenges, including wire displacement, difficulty in discerning the position of the tip of the wire intraoperatively, and in some cases a long distance between wire entry point and lesion^1^. These make optimal incision placement a challenge and lead to extensive dissection to remove the target lesion, and potential failure of localisation. Wire placement occurs on the day of surgery, which can cause logistic issues for radiology and surgery departments, and lead to delays in the operating theatre. However, wires remains the default method of localization due to the limitations of other methods of localization and given the long term data supporting its effectiveness^2^.

Iodine (^125^I) radioactive seed localization has been used in some units, and has some logistical advantages^3^, however, the radiation safety precautions required to set up and support this service limits its widespread implementation^4^. Radio-occult lesion localization (ROLL) has also been used, but offers less logistical advantage compared to seeds, as it still requires radioisotope injection to occur within 24 hours of surgery. Unless contrast is also given, ROLL does not offer the surgeon mammographic or ultrasound confirmation of the site of injection in relation to the lesion^5^. Radioactive seed localization and ROLL are equally reliable to wire localization^2^.

As an alternative to wire localization, the US Food and Drug Administration (FDA) approved a new device for localizing nonpalpable breast lesions in 2014. Savi Scout® (Cianna Medical, Merit Medical Systems, Inc. South Jordan, UT) is a wireless, nonradioactive, wave reflection implant system that enables surgeons to safely and accurately remove breast tissue. The implantable 12 x 1.6mm electromagnetic wave reflector is inserted into the breast lesion under radiological guidance. The SSL system involves the insertion of the electromagnetic wave reflector into the target tissue using a sterile 16-gauge introducer needle delivery system (available needle lengths of 5, 7.5 and 10 cm) under mammogram or ultrasound guidance. The reflector is activated by infrared light impulses generated by the console probe and uses two antennas to reflect an electromagnetic wave signal back to the handpiece. The signal is processed by the console to produce an audible and visual distance to target feedback to the operator, guiding the removal of the lesion throughout surgical dissection. The detector has “insignificant MRI bloom”, displays the real-time measurement of lesion location during use, and has an alleged accuracy of ±1 mm (Cianna Medical). Savi Scout has been used in US sites since its FDA approval with several publications outlining its efficacy. Tingen et al 2020 published a retrospective review of single institution cases comparing 175 wire localisations and 320 Savi Scout ®. They have shown Savi Scout ® to be effective at localising lesions with a lower re-excision rate in the Savi Scout ® group vs the wire (5.3% vs 13.7%). These single institution series are useful but are prone to bias and limited in their usefulness for adoption of the technique amongst inexperienced users. Savi Scout® was CE marked for use in Europe in 2020 and early data is emerging from European users^6^. Systematic review of 11 studies of Savi Scout® includes 842 Savi Scout® reflectors^6^. Of these, 839 were successfully placed and 836 were successfully retrieved using SSL. This gives a successful deployment rate of 99.64% and a successful retrieval rate of 99.4%. Of the 839 successfully placed reflectors, 624 were inserted in malignant lesions with 80 requiring re-excision. The re-excision rate was therefore 12.8%. Across the four studies directly comparing Savi Scout® to wire-guided localisation, 545 WGLs were performed, and 264 reflectors were placed to localise malignant lesions. Of these, 115 WGLs required re-excision compared to 34 SSLs. This gives a re-excision rate of 21.1% for WGL and 12.9% for SSL. A chi-square test found this difference to be statistically significant (χ2 with Yates' correction=7.4639, p<0.01). This emerging data demonstrates that Savi Scout® can be used to localise breast lesions and may reduce re-excision rates. The quality of the systematic review is dependent on the quality of the datasets. We know from the introduction of any new device that it takes time for the team to learn and adopt and to get the best out of the device. Prospective data collection amongst new sites enables us to collate accurate information on outcomes and compare to existing technologies and to enable learning as a group. Larger data sets also enable us to limit the effect of confounders and bias and the limitations of retrospective data collection.

Savi Scout® is also being used for axillary nodal identification, with one published study of 23 patients demonstrating feasibility with a nodal retrieval rate of 100%^7^.

1.2. RATIONALE FOR PERFORMING THE STUDY

This study is being performed to evaluate the efficacy of Savi Scout® in localising non-palpable breast lesion and axillary marking. The majority of studies of evaluating Savi Scout® localisation in impalpable breast lesions have had small and retrospective in design. Hence the use of a prospective large multi-centre cohort enables analysis of the benefits of this usage of Savi Scout® with a higher level of evidence . De-escalation of axillary surgery reduces treatment morbidity but depends on effective methods of nodal localisation^8-13^. There is very limited evidence for the use of Savi Scout® in axillary nodal identification. A large prospective dataset will assist in appraisal of the efficacy of Savi Scout® for this purpose.

**2. HYPOTHESIS**

Savi Scout® is an accurate, safe method of localising impalpable lesions and axillary nodes.

**3. STUDY OBJECTIVES / AIMS**

3.1. PRIMARY OBJECTIVES

- - 1. To evaluate the efficacy of localization of non-palpable breast lesions with Savi Scout ®.
    2. To establish the efficacy of axillary node marking with Savi Scout ®.

3.2. SECONDARY OBJECTIVES

To compare the efficacy of Savi Scout ® with hookwire locations of non-palpable breast lesions.

**4. STUDY DESIGN**

4.1. DESIGN / STUDY TYPE

The study will be a prospective audit of outcomes related to localisation with Savi Scout ®.

4.2. EXPECTED PARTICIPANT NUMBERS

1000 globally, 50 from Chris O’Brien Lifehouse

4.3. TIME PERIOD OF THE STUDY

*:*

| **Task** | **Start Date** | **End Date** |
| --- | --- | --- |
| **Ethics Submission** | May 2023 | May 2023 |
| **Ethics Review and Approval** | Mid-May 2023 | June 2023 |
| **Advertising** | June 2023 | July 2023 |
| **Recruitment** | July 2023 | July 2024 |
| **Collection of data** | July 2023 | July 2024 |
| **Analysis of Data** | August 2024 | Sep 2024 |
| **Preparations of Reports** | Jan 2025 | Feb 2025 |
| **Publication Draft** | March 2025 | March 2025 |
| **Submission of Publications and Final Reports** | May 2025 | June 2025 |

4.4 ENDPOINTS

PRIMARY ENDPOINTS

Savi Scout ®is an accurate method of identifying non-palpable breast lesion.

SECONDARY ENDPOINTS

Savi Scout ®is an accurate method of identifying axillary nodes.

4.5. CENTRES (STUDY SITES)

| **Site Name/s** | Chris O’Brien Lifehouse |
| --- | --- |
| **Site Contact/Investigator** | A/Prof Cindy Mak |
| **Public Health Organisation (PHO)** | **YES** |
| **Study Procedures** | Data Collection |

**5. STUDY PARTICIPANTS**

5.1. INCLUSION CRITERIA

Patients undergoing a breast conserving localization procedure or axillary nodal marking using the Savi Scout ® System.

18 years of age

Willing to participate in the study.

5.2. EXCLUSION CRITERIA

Pregnancy

Unable to read and write English to a level required for consent and completion of written questionnaires.

Unwilling to provide consent.

5.3 KEY ELEMENTS OF RECRUITMENT (AS PER NS)

1. Participants who have decided to have their localisation carried out with Savi Scout will be recruited for the study.

2. Participants will be identified by treating clinician. They will be given the option to have information regarding their localisation included into the prospective database.

3. There will be no impact in the relationship between researcher and potential participant as the participant will go ahead with their procedure regardless of their involvement in the study.

4. The participants will be verbally informed of what the study entails and they will be given a patient information sheet prior to making their decision. The participant will be able to ask questions from the researcher after having time to read the patient information sheet.

5. Given that this is audit of information regarding their treatment, there is no risk to the patient or for the viability of the project.

5.4 CONFOUNDERS

5.5 STUDY LIMITATIONS

Given that this is a multi-centre study, there will be differences in the radiologist’s technique in placing the Savi Scout® localisation device and the surgeon’s technique in retrieving the targeted lesion.

This may mean that if a particular group has a large contribution to the dataset, the results may be skewed.

This study is a prospective audit and does not have the same level of evidence as a randomised controlled trial.

**6. STUDY PROCEDURES**

6.1. INVESTIGATION PLAN

The multicentre component of the trial involves a prospective audit of information relating to the use of Savi Scout®.

This involves documenting this data into a secure web-based Redcap database.

The dataset collected is the same from every international site.

There is no intervention that is carried out for the study as this is an audit.

The procedures related to Savi Scout® placement are part of normal practice and are not influenced by this study given the collection of data occurs after the decision to use Savi Scout®. Savi Scout® is an established technology for localisation of impalpable breast lesions and axillary nodes at Chris O’Brien Lifehouse.

This audit will have data contributed from multiple sites globally. The information collected will investigate the margin status of excision, accuracy of placement, timing of the placement of Savi Scout®, and the proportion of Savi Scout® placed at the time of biopsy, pathological weight of specimen, size of MRI bloom around Savi Scout® , transcutaneous detection rate, reoperation rate, complications, cancellation rate on day of surgery, reason for cancellation on day of surgery, time of day of start of surgery and identification rate of axillary nodes using Savi Scout.

Comparative analysis will be performed with the same parameters for participants that have undergone hookwire-localisation of impalpable breast lesions. Information of the hookwire-localisation cohort have been recruited from Manchester University NHS Foundation Trust/Manchester University.

6.2. INFORMATION AND CONSENT (OR WAIVER OF CONSENT)

Those who meet the eligibility criteria will be provided with a Patient Information Sheet during one of their routine consultations with their treating surgeon. The participant will have the opportunity to discuss any questions with their surgeon before giving their consent. Those who are agreeable to participate will be asked to complete a hardcopy consent form prior to data being entered into the database. Participants would have time from their initial consult to the time of surgery which is generally 1 week to provide consent. The time to give consent will not be less than 24 hours.

**7. OUTCOMES**

7.1. DEFINITION OF OUTCOMES

Outcome measures that are being assessed relate to the efficacy of Savi Scout® in localising non-palpable breast lesions and axillary lymph nodes.

**8. STATISTICAL CONSIDERATIONS**

8.1. SAMPLE SIZE OR POWER CALCULATION

Power Calculation - with n=1000 patients per group, the upper limit of the observed one-sided 95% confidence interval for the difference between failure rates (seed vs wire) is expected to be less than 0.9% with 80% power, assuming the two methods both have an expected failure rate of 0.6%. Hence, if a 0.9% difference is considered an acceptable equivalence margin (eg. 0.6% for wire and 1.5% for seed), 1000 patients per group should be sufficient to establish equivalence.

8.2. PROVIDE A DETAILED ANALYSIS PLAN

Simple summary statistics will be calculated for each outcome and regression analysis used to control for predictive variables. Data will be tested for distribution and differences between groups using unpaired t-tests, Mann-Whitney U tests and Chi squared tests as appropriate, based on recruitment.

**9. DATA COLLECTION AND CONFIDENTIALITY AND STORAGE AND ARCHIVING OF STUDY**

Confidentiality, storage and archiving will be conducted according to Australian and NSW Research Standards.

Each participant will be given a unique study identification code and data will be entered and stored using this code. This will be used to collate their data in a de-identified manner and link this data to their clinical history. Data will be collected on the electronic REDCap system, hosted by Manchester University NHS Foundation Trust/Manchester University. REDCap is a secure, web-based application designed exclusively to support data capture for research studies REDCap provides: 1) an intuitive interface for data entry (with data validation); 2) audit trails for tracking data manipulation and export procedures; 3) automated export procedures for seamless data downloads to common statistical packages (SPSS, SAS, Stata, R); and 4) procedures for importing data from external sources.

REDCap is developed and maintained by a team at the Vanderbilt University and licensed free of charge. Access to a REDCap database is restricted and password protected. Only the study investigators will have access to completed consent data and research data. In this study the REDCap database is designed by Vanderbilt University and hosted by Manchester University NHS Foundation Trust/Manchester University.

For analysis, the deidentified participant data will be exported from REDCap at Manchester University for statistical analysis.

The CPI is the administrator of the account and will be the custodian of the data. Ownership and access will be restricted to active Investigators on the Project. When an Investigator is no longer active their access will be deactivated, and Ethics will be notified. If the CPI is changed then Ethics will be notified.

9.1. FORMS AND PROCEDURE FOR COLLECTING DATA

Data will be collected via the REDCap system and the CRF that is attached identifies the data fields for data collection.

There will be no images collected for this study.

Artificial intelligence will not be used for data collection in the study.

9.2. SYSTEMS

REDCap has been disseminated for local use at more than 1,005 other academic/non-profit consortium partners in 79 countries. Vanderbilt leads the REDCap Consortium, which currently supports more than 99,000 projects and 128,000 users. More information about the consortium and system security can be found at http://www.projectredcap.org/. REDCap is a secure, web-based application designed to support data capture for research studies, providing 1) an intuitive interface for validated data entry; 2) audit trails for tracking data manipulation and export procedures; 3) automated export procedures for seamless data downloads to common statistical packages; and 4) procedures for importing data from external sources.

REDCap is created by Vanderbilt University, with the server hosted by the University of Manchester. REDCap was developed specifically around HIPAA-Security guidelines. Web browser communication to the server is SSL-encrypted by default. All other ports are firewall protected. Data is stored in MySQL databases on a separate server. This server is behind a firewall and can only be accessed from the IP address of the web server. An SSL- tunnel encrypts communication between the web and databases servers. File upload is secured between servers using theWebDAV protocol with SSL. "At rest" encryption is in place on the database server (aes-xts-plain64:sha256 with 512-bit keys). Daily back-ups are made of both servers and stored for two weeks prior to being deleted. Operating security updates are installed automatically. Antivirus software runs to a scheduled protocol on the web server. User passwords are managed directly. Accounts are disabled after 5 failed login attempts. Users are auto logged out after 30 mins of no activity. Users are forced to change password after 90 days. Daily audit tracking of users is in place with removal of unused user accounts. REDCap servers are housed at the University of Manchester and all web-based information transmission is encrypted.

Data collection will occur in accordance with Caldicott II principles. Data for each patient will be anonymised using a unique alphanumeric study identification code. No patient identifiable data will be recorded for the purpose of the audit.

9.3 RESEARCH DATA MANAGEMENT PLAN (RDMP)

Research data management plan is attached to the ethics submission.

**10. ETHICS AND PROTOCOL AMENDMENTS**

**11. PUBLICATION & INTELLECTUAL PROPERTY**

This is a collaborative study and hence multiple sites will have site specific data as per the overall trial design. Local collaboratives and hospital Trusts will have ownership of their own data and will be able to present it locally if they wish.

Publication and dissemination of data will be through international conference presentation and peer reviewed journals. International presentations and publications will be made on behalf of the IBRA-net Study Group.

Three levels of authorship are proposed based on degree of study participation:

Named authors will be required to meet the International Committee of Medical Journal Editors (ICMJE) criteria (www.icmje.org) for authorship based on the following four criteria:

1. Substantial contribution to the conception or design of the work; or the acquisition, analysis or interpretation of the data for the work and

2. Drafting the work or revising it critically for important intellectual content and

3. Final approval of the version to be published and

4. Agreement to be accountable for all aspects of the work in ensuring that questions related to the accuracy or integrity of any part of the work are appropriately investigated and resolved.

All citable collaborators will be listed at the end of the paper and their roles identified. Collaborators may be invited to sit on the iBRA-net Writing Group which will be responsible for drafting manuscripts and preparing them for publication.

Citable collaborators will have made a considerable contribution to the study but will not have met the ICMJE criteria (listed above) for authorship. These will include study leads at each centre and other team members who have recruited at least 10 patients to the study.

Final reports will be prepared in accordance with the STROBE (Strengthening the Reporting of Observational Studies in Epidemiology) guidelines.

**12. REFERENCES**

1. Rahusen FD, Bremers AJ, Fabry HF, et al. Ultrasound-guided lumpectomy of nonpalpable breast cancer versus wire-guided resection: a randomized clinical trial. *Ann Surg Oncol* 2002;9(10):994-8. doi: 10.1007/bf02574518 [published Online First: 2002/12/05]

2. Chan BK, Wiseberg-Firtell JA, Jois RH, et al. Localization techniques for guided surgical excision of non-palpable breast lesions. *Cochrane Database Syst Rev* 2015;2015(12):Cd009206. doi: 10.1002/14651858.CD009206.pub2 [published Online First: 2016/01/01]

3. Langhans L, Tvedskov TF, Klausen TL, et al. Radioactive Seed Localization or Wire-guided Localization of Nonpalpable Invasive and In Situ Breast Cancer: A Randomized, Multicenter, Open-label Trial. *Ann Surg* 2017;266(1):29-35. doi: 10.1097/sla.0000000000002101 [published Online First: 2017/03/04]

4. NABON Breast Cancer Audit

(NBCA) Jaarrapportage NBCA 2014 [Available from: <https://dica.nl/jaarrapportage-2014/> accessed 25/04 2023.

5. Rampaul RS, Bagnall M, Burrell H, et al. Randomized clinical trial comparing radioisotope occult lesion localization and wire-guided excision for biopsy of occult breast lesions. *Br J Surg* 2004;91(12):1575-7. doi: 10.1002/bjs.4801 [published Online First: 2004/10/27]

6. Kasem I, Mokbel K. Savi Scout® Radar Localisation of Non-palpable Breast Lesions: Systematic Review and Pooled Analysis of 842 Cases. *Anticancer Res* 2020;40(7):3633-43. doi: 10.21873/anticanres.14352 [published Online First: 2020/07/06]

7. Baker JL, Haji F, Kusske AM, et al. SAVI SCOUT® localization of metastatic axillary lymph node prior to neoadjuvant chemotherapy for targeted axillary dissection: a pilot study. *Breast Cancer Res Treat* 2022;191(1):107-14. doi: 10.1007/s10549-021-06416-z [published Online First: 2021/10/16]

8. Krag DN, Anderson SJ, Julian TB, et al. Sentinel-lymph-node resection compared with conventional axillary-lymph-node dissection in clinically node-negative patients with breast cancer: overall survival findings from the NSABP B-32 randomised phase 3 trial. *Lancet Oncol* 2010;11(10):927-33. doi: 10.1016/s1470-2045(10)70207-2 [published Online First: 2010/09/25]

9. Mansel RE, Fallowfield L, Kissin M, et al. Randomized multicenter trial of sentinel node biopsy versus standard axillary treatment in operable breast cancer: the ALMANAC Trial. *J Natl Cancer Inst* 2006;98(9):599-609. doi: 10.1093/jnci/djj158 [published Online First: 2006/05/04]

10. Donker M, van Tienhoven G, Straver ME, et al. Radiotherapy or surgery of the axilla after a positive sentinel node in breast cancer (EORTC 10981-22023 AMAROS): a randomised, multicentre, open-label, phase 3 non-inferiority trial. *Lancet Oncol* 2014;15(12):1303-10. doi: 10.1016/s1470-2045(14)70460-7 [published Online First: 2014/12/03]

11. Kuehn T, Bauerfeind I, Fehm T, et al. Sentinel-lymph-node biopsy in patients with breast cancer before and after neoadjuvant chemotherapy (SENTINA): a prospective, multicentre cohort study. *Lancet Oncol* 2013;14(7):609-18. doi: 10.1016/s1470-2045(13)70166-9 [published Online First: 2013/05/21]

12. Caudle AS, Yang WT, Krishnamurthy S, et al. Improved Axillary Evaluation Following Neoadjuvant Therapy for Patients With Node-Positive Breast Cancer Using Selective Evaluation of Clipped Nodes: Implementation of Targeted Axillary Dissection. *J Clin Oncol* 2016;34(10):1072-8. doi: 10.1200/jco.2015.64.0094 [published Online First: 2016/01/27]

13. Goyal A, Cramp S, Marshall A, et al. ATNEC: A multicenter, randomized trial investigating whether axillary treatment can be avoided in patients with T1-3N1M0 breast cancer with no residual cancer in the lymph glands after neoadjuvant chemotherapy. *Journal of Clinical Oncology* 2022;40(16_suppl):TPS615-TPS15. doi: 10.1200/JCO.2022.40.16_suppl.TPS615

**13. APPENDICES**
